# Effect of Chronic Athletic Activity on Brown Fat in Young Women

**DOI:** 10.1371/journal.pone.0156353

**Published:** 2016-05-31

**Authors:** Vibha Singhal, Giovana D. Maffazioli, Kate E. Ackerman, Hang Lee, Elisa F. Elia, Ryan Woolley, Gerald Kolodny, Aaron M. Cypess, Madhusmita Misra

**Affiliations:** 1 Pediatric Endocrine Unit, Massachusetts General Hospital and Harvard Medical School Boston, Massachusetts, United States of America; 2 Neuroendocrine Unit, Massachusetts General Hospital and Harvard Medical School, Boston, Massachusetts, United States of America; 3 Division of Sports Medicine, Boston Children's Hospital and Harvard Medical School, Boston, Massachusetts, United States of America; 4 Department of Biostatistics, Massachusetts General Hospital and Harvard Medical School, Boston, Massachusetts, United States of America; 5 Division of Nuclear Medicine and Molecular Imaging, Department of Radiology, Beth Israel Deaconess Medical Center and Harvard Medical School, Boston, Massachusetts, United States of America; 6 Diabetes, Endocrinology, and Obesity Branch, NIDDK, NIH, Bethesda, Maryland, United States of America; University of Sydney, AUSTRALIA

## Abstract

**Background:**

The effect of chronic exercise activity on brown adipose tissue (BAT) is not clear, with some studies showing positive and others showing negative associations. Chronic exercise is associated with increased resting energy expenditure (REE) secondary to increased lean mass and a probable increase in BAT. Many athletes are in a state of relative energy deficit suggested by lower fat mass and hypothalamic amenorrhea. States of severe energy deficit such as anorexia nervosa are associated with reduced BAT. There are no data regarding the impact of chronic exercise activity on BAT volume or activity in young women and it is unclear whether relative energy deficiency modifies the effects of exercise on BAT.

**Purpose:**

We assessed cold induced BAT volume and activity in young female athletes compared with non-athletes, and further evaluated associations of BAT with measures of REE, body composition and menstrual status.

**Methods:**

The protocol was approved by our Institutional Review Board. Written informed consent was obtained from all participants prior to study initiation. This was a cross-sectional study of 24 women (16 athletes and8 non-athletes) between 18–25 years of age. Athletes were either oligo-amenorrheic (n = 8) or eumenorrheic (n = 8).We used PET/CT scans to determine cold induced BAT activity, VMAX Encore 29 metabolic cart to obtain measures of REE, and DXA for body composition.

**Results:**

Athletes and non-athletes did not differ for age or BMI. Compared with non-athletes, athletes had lower percent body fat (p = 0.002), higher percent lean mass (p = 0.01) and trended higher in REE (p = 0.09). BAT volume and activity in athletes trended lower than in non-athletes (p = 0.06; p = 0.07, respectively). We found negative associations of BAT activity with duration of amenorrhea (r = -0.46, p = 0.02).BAT volume correlated inversely with lean mass (r = -0.46, p = 0.02), and positively with percent body fat, irisin and thyroid hormones.

**Conclusions:**

Our study shows a trend for lower BAT in young female athletes compared with non-athletes, and shows associations of brown fat with menstrual status and body composition. Brown fat may undergo adaptive reductions with increasing energy deficit.

## Introduction

Brown adipose tissue (BAT) and lean mass are implicated in energy homeostasis and are present in variable amounts in humans. Detectable BAT activity is reported to be lower with increasing age [1, 2] and BMI [3], and is upregulated by thyroid hormones [4]. In addition, estrogen upregulates bone morphogenetic protein 8 (BMP8) which induces BAT in rodents [5].Another potential up regulator of BAT activity in humans is habitual physical activity [6], potentially contributing to the increased resting energy expenditure (REE) seen in athletes. In contrast, BAT activity may be lower in ‘hyperexercising’ athletes as an adaptation to conserve energy, as has been recently reported in male endurance athletes [7].

BAT activity varies with metabolic state, and is lower when subjects are in a state of energy surplus (obesity) [2], suggesting that lower BAT activity may contribute to obesity through reduced non-shivering thermogenesis. Conversely, BAT activity is low in male endurance athletes [7] and in anorexia nervosa, a state of extreme energy deficit and hypogonadism, likely as an adaptive mechanism to conserve energy [8]. Of course, anorexia nervosa is associated with alterations in many hormonal axes, which may also impact BAT volume and activity. Many athletes (particularly if amenorrheic) are believed to be in a state of relative energy deficit [9], and are a unique model of intense exercise, energy deficiency and hypogonadism. In contrast, eumenorrheic athletes represent a state of intense exercise, variable energy status, and normal estrogen levels [10].Thus far, studies have not examined the relative impact of chronic exercise versus chronic estrogen deficiency on BAT activity.

Based on data in women with anorexia nervosa [8] and male endurance athletes [7]we hypothesized that young hyperexercising female athletes would overall have lower BAT activity compared with normal-weight non-athletes in an attempt to preserve energy. We thus examined cold-induced brown fat volume and activity in young female athletes 18–25 years old compared to non-athletes in a similar age and weight range. We also examined associations of BAT with measures of resting energy expenditure, body composition, and duration of amenorrhea. We hypothesized that chronic hypoestrogenism will be negatively correlated with BAT.

## Subjects and Methods

### Subjects

The protocol was approved by the Institutional Review Board of Partners Health Care. Written informed consent was obtained from all participants prior to study initiation. We studied 25 young women (16 athletes and 9 non-athletes) between 18–25 years of age. In the athlete group, subjects were categorized as oligo-amenorrheic (n = 8) if they had absence of menses for ≥ three months within a period of oligomenorrhea (cycle length>six weeks) for the previous six months, after other causes of amenorrhea were ruled out. Eumenorrheic athletes and non-athletes were required to have ≥nine menses in the preceding year. Duration of amenorrhea was assessed as the number of months of missed menses after the first day of the last menstrual cycle. We enrolled athletes who engaged in weight-bearing aerobic activities involving the legs (such as track, cross-country, soccer and field hockey) for ≥ 4 hours/week and/or who ran ≥ 20 miles/week for at least 6 months in the year preceding the study. Non-athletes were required to be engaged in < 2 hours per week of weight-bearing exercise activity. All participants were between the 10th and 85th percentiles for BMI for age. Exclusion criteria included conditions other than endurance training that may cause amenorrhea such as pregnancy, thyroid dysfunction, primary ovarian insufficiency, polycystic ovarian disease, hyperprolactinemia and active anorexia nervosa (determined by the study psychologist). Subjects were excluded if they had used medications that may influence brown fat activity:β-adrenergic agonists, β-blockers, estrogen-progesterone combination pills and benzodiazepines in the three months preceding the study. All participants were recruited from the community through advertisements and referrals from healthcare providers throughout the year.

### Study Design

This was a cross-sectional study in which all subjects completed a medical history, physical examination and anthropometric measurements [weight, height, body mass index (BMI)] during a single visit at the Clinical Research Center of our institution. Based on previous studies published by our group, we estimated that a sample size of 23 would detect a difference of 50% in BAT positivity between athletes and normal-weight non-athletes, assuming 80% positivity for BAT in healthy women [11].

#### Brown Adipose Tissue Assessment

Cold induced BAT activity and BAT volume were obtained through fluoro-deoxy-glucose (FDG) positron emission tomography/computed tomography (PET/CT) scan. All subjects fasted overnight and did not participate in any rigorous physical activity for at least 12 hours before the study visit. Subjects wore a cooling vest at 14°C for 120 min before the PET/CT scan and the temperature was not altered regardless of shivering [12]. BAT activity was quantified by 18F-FDG uptake in the cervical-thoracic depots, which have been shown to be the most frequent region to detect BAT, and represent 90–100% of total detectable BAT. A dose between 5–8 mCi of 18F-FDG was injected, and ImageJ 1.5e, Wayne Rasband software at the Beth Israel Deaconess Medical Center nuclear medicine viewing and analysis shareware, was used for analysis. There is no current consensus in the literature regarding which measure of brown fat (total volume or mean standardized uptake value in a single volume of interest: SUV mean) is more reliable [13], and therefore both parameters were analyzed. We calculated the sum of the following product: (SUVmean * BAT volume) seen in each voxel containing BAT. We have called this value “BAT activity”, and this is the “total body BAT glucose metabolism”. We found a strong correlation between two measures (brown fat volume and BAT activity: r = 0.82, p<0.0001) and thus report both. Based on previous studies [14, 15], we used a threshold of 1.0 for SUV parameters to segregate BAT and white adipose tissue (WAT).BAT estimations were done by a single investigator who was blinded to the group allocation of the subject. The intra observer coefficient of variation was 3.1% for total volume and 0.46% for SUV mean. Quantification was performed by drawing an ROI (Region of Interest) on a slice-by-slice axial basis covering the entire cervico-thoracic area regardless of the visual impression of positivity or negativity on PET/CT. In order to quantify only brown fat, two criteria were applied: only pixels with SUV max between 1 and 30 and CT Hounsfield Units that describe fat (-250 to -10 HU) were included. Pixels within the drawn area that met both criteria for BAT were included in the volume and SUV mean quantification.

Muscle FDG uptake was quantified by SUV mean. Measurements were obtained by drawing an ROI in the medial portion of the sternocleidomastoid (SCM) muscle of each patient while avoiding any surrounding BAT activity if present. In one case, the large amount of FDG uptake in BAT surrounding the SCM limited the quantification in the region so the ROI was drawn in the trapezoid muscle. None of the subjects demonstrated increased muscular glucose uptake or altered biodistribution of 18F-FDG.

Our scanner and software report BAT volume by normalizing the amount of FDG uptake in each voxel to a subject’s total body weight. Because there is very limited uptake of FDG in white adipose tissue (WAT), we only corrected BAT volume to the proportion of lean body mass (LBM) as determined by DXA: BAT volume (mL) * [(subject’s LBM in g)/(population LBM in g)]. We call this BAT vol adjusted for LBM. This adjustment leads to a comparatively higher BAT volume per voxel in the subjects who have the highest LBM.

#### Measures of Resting Energy Expenditure and Body Composition

Fasting resting energy expenditure (REE) was performed under thermal neutrality using VMAX Encore 29 metabolic cart (Viasys Healthcare, Carefusion; San Diego, CA). Subjects did not alter their usual food intake on the days preceding the study visit. Measures of body composition were determined using dual x-ray absorptiometry (DXA) (Hologic QDR-Discovery A, apex software version 13.3; Hologic Inc, Waltham, MA).

#### Biochemical Analysis

Thyroid stimulating-hormone (TSH), total triiodothyronine (total T3) and free thyroxine (free T4) were assessed by LabCorp using standard immunoassays (Roche Cobas E601® Immunology Analyzer). Irisin was measured using an ELISA (Adipogen; Liestal, Switzerland). Intra-assay coefficient of variation (CV) was 6.9% and the detection limit was 0.001 mcg/mL We recommend caution in interpreting the irisin results as there is a controversy about the validity of the commercially available kits in evaluating irisin [16]. Instead of measuring leptin, we used total fat mass as a surrogate for leptin levels, given the very strong correlations of leptin with fat mass [17, 18]. Further, instead of measuring estradiol levels, which vary across a menstrual cycle and represent levels at a single moment in time, we used duration of amenorrhea as a measure of chronic estrogen deficiency.

#### Statistical Analysis

We used JMP Statistical version 10.0 (SAS institute, Inc., Cary, NC) for all analyses. Demographics, clinical, biochemical and brown fat characteristics within each group were summarized as means±SD. The between group statistical comparisons for continuous outcomes were performed by using independent sample T-tests or Wilcoxon Rank-Sum tests depending on the distribution of the analyzed variables. Brown fat volume and SUV mean were logarithmically transformed prior to analyses to approximate a normal distribution. Fisher’s Exact Test was used for categorical variables. To examine linear associations of brown fat volume with clinical and biochemical characteristics, we used the Pearson’s correlation coefficient(r) for normally distributed variables, and Spearman’s rank correlation coefficient (ρ) for non-normally distributed variables. We estimated correlations of BAT activity and volume with clinical and biochemical characteristics for all subjects taken together. We also applied multivariate modeling when necessary to control for covariates. A p-value of less than 0.05 was considered significant.

## Results

### Participants Characteristics

Clinical characteristics of subjects (athletes vs. non-athletes) are summarized in Table 1. Athletes and non-athletes did not differ for age, menarchal age or BMI. By study design, hours/week of physical activity was higher in athletes. Heart rate and temperature were lower in athletes. Similarly, total fat mass and percentage of body fat were lower in athletes as compared to non-athletes. REE and total lean mass trended higher, and percentage of lean mass was higher in athletes compared to non-athletes. Athletes and non-athletes did not differ for caloric intake, irisin or thyroid hormone levels. There was no significant difference in the season of evaluation between athletes vs. non-athletes.

**Table 1:** Baseline and Brown Fat Characteristics Athletes and Non-athletes (NA).

Characteristics	Athletes (n = 16)	Non-athletes (n = 8)	P value
Age (years)	21.7±2.2	21.6±2.1	0.86
Age at menarche (years)	12.7±2.2	12.4±0.9	1.00*
Height (cm)	163.1±4.5	164.4±9.8	0.64
Weight (kg)	57.8±7.4	61.7±12.6	0.34
BMI (kg/m^2^)	21.8±2.6	22.6±2.4	0.42
Activity (hours/week)	11.3±5.4	0.6±0.5	**0.0001**
Systolic Blood Pressure (mmHg)	92.8±20.2	99.6±12.7	0.67*
Diastolic Blood Pressure (mmHg)	59.2±17.2	62.2±13.0	0.86*
Heart Rate (bpm)	59.3±7.9	73.3±10.3	**0.001**
Temperature (°C)	36.4±0.6	36.8±0.5	**0.03**
Percent Body Fat	24.5±5.7	33.0±5.7	**0.002**
Total Fat Mass (kg)	14.8±4.9	20.8±5.9	**0.01**
Percent Lean Mass	71.8±5.5	64.4±5.9	**0.01**
Total Lean Mass (kg)	42.2±3.7	40.2±9.3	0.08*
Resting Energy Expenditure (REE) (cal)	1183±116	1067±121	0.09
REE/Total Lean Mass (Cal/kg)	0.031± 0.002	0.029±0.004	0.39
Irisin (mcg/ml)	3.11±0.87	3.40±0.80	0.44
TSH (microU/mL)	1.9±0.8	1.46±0.7	0.26
Total T3 (ng/dL)	91.9±20.5	103.4±8.7	0.17
Free T4 (ng/dL)	0.9±0.1	1.0±0.1	0.21
**Brown Fat**			
Visual BAT positivity (n)	6	6	0.19**
Log BAT volume (mL)	0.91±0.66	1.45±0.58	0.06
Log BAT volume adjusted for lean mass (mL)	1.86±1.87	3.27±1.34	0.07
SUV mean (g/mL)	1.53±1.84	1.95±1.44	0.07*

Data presented as mean± SD. Significant p values are bolded.

NA: non-athletes SUV mean: mean standardized uptake value

The independent samples t-test used for two group comparisons for normally distributed outcomes.

* The Wilcoxon rank sum test was used for two group comparisons of the outcomes that were not normally distributed.

**Fisher´s Exact Test.

### Brown Fat Estimation

**Fig 1:**
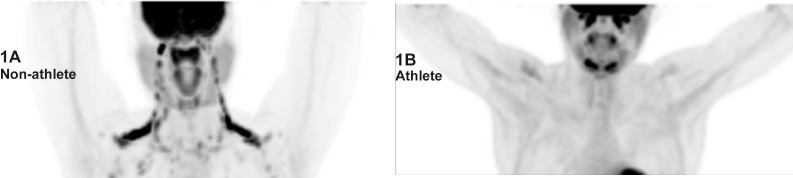
Brown Fat Activity Differences between Athletes and Non-Athletes. **(1A)** Maximum Intensity Projection of an 18F-FDG PET-CT scan performed in a non-athlete shows multiple and bilateral foci of increased glucose uptake along the laterocervical, supraclavicular, axillary regions, corresponding to metabolically active BAT in these regions. (**1B)** Maximum Intensity Projection of an 18F-FDG whole body PET-CT scan performed in an athlete. Physiological glucose uptake is seen in the brain, salivary glands and myocardium. No active BAT is seen in this patient.

**Fig 2:**
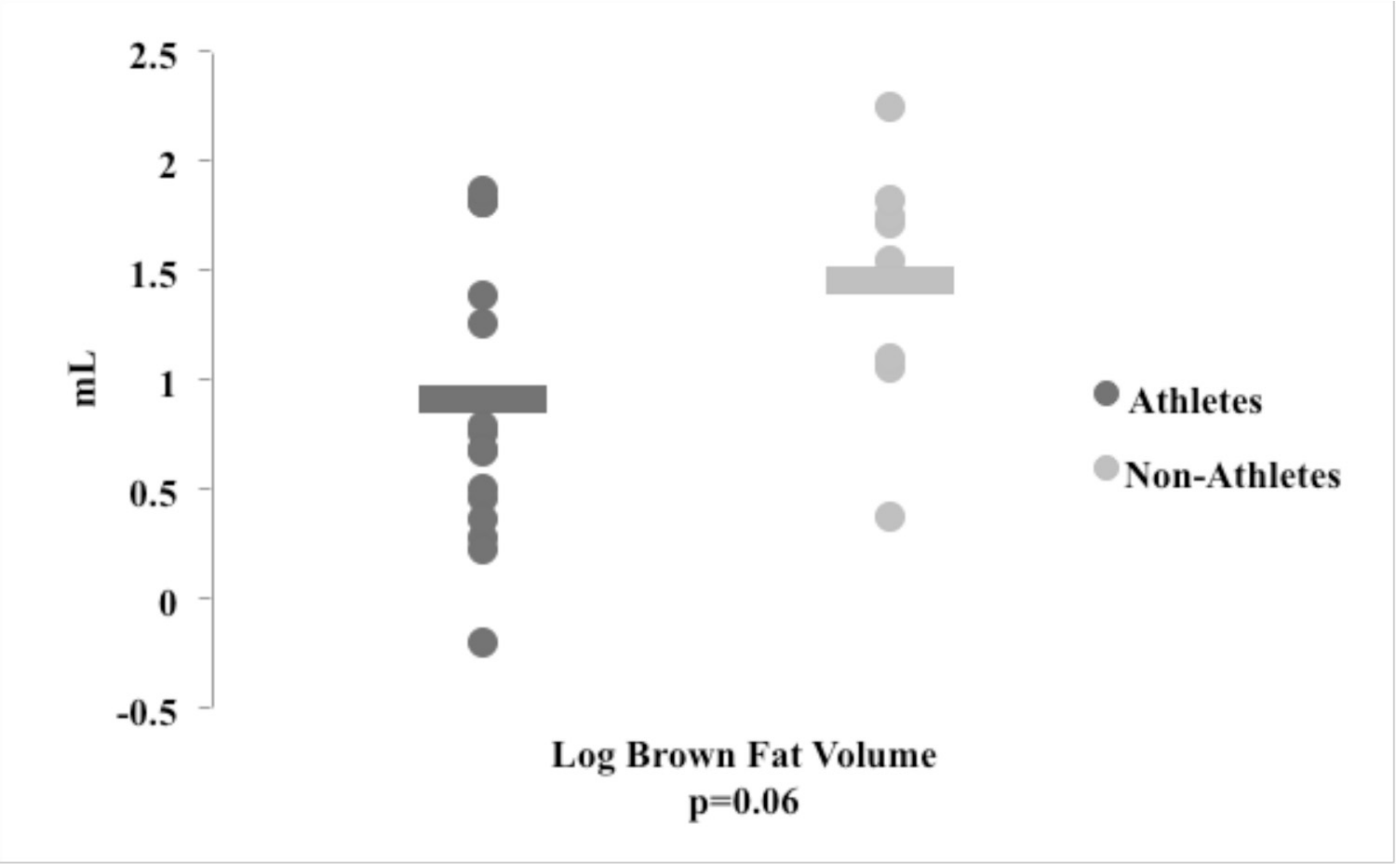
Brown Fat Volume Differences between Athletes and Non-Athletes. Brown fat volume trended lower in athletes (dark grey) compared to non-athletes (light grey).

Fig 1 shows FDG uptake indicative of visual BAT positivity in one representative athlete and one non-athlete. We found no statistically significant difference between athletes and non-athletes in the proportion of participants with visual BAT positivity (37.5% vs.75% p = 0.19), though the proportion of athletes with visual BAT was only half that in non-athletes. When BAT was quantified, brown fat volume trended lower in athletes than in non-athletes (p = 0.06) (Fig 2).This trend was maintained after we normalized brown fat volume for lean body mass (Table 1). Within athletes, we found no impact of menstrual status, when comparing oligo-amenorrheic athletes vs. eumenorrheic athletes for BAT measures (BAT volume: 0.82±0.71 vs. 1.00±0.65 mL, p = 0.62and activity1.05±1.07 vs. 2.01±2.36 g/mL, p = 0.34). However when we assessed log BAT volume after adjusting for lean body mass (as higher lean body mass can cause greater BAT), we found a trend towards lower BAT volume in oligoamenorrheic athletes (n = 8) compared to all eumenorrheic athletes and non-athletes (n = 16) (1.43±0.61 vs. 2.78 ±0.43 ml, p = 0.08).

### Relationship between Clinical Characteristics and Brown Fat Measures (all subjects)

Linear associations between clinical characteristics and brown fat parameters for all subjects taken together are shown in Table 2. We found no associations of BAT measures with age or BMI; however, BAT volume correlated positively with heart rate and body temperature. We found a negative linear association between BAT activity and duration of amenorrhea (r = -0.46, p = 0.02), which persisted after controlling for lean mass (β estimate -0.22, p = 0.03).

**Table 2:** Associations of Brown Fat with Clinical and Biochemical Characteristics.

All Subjects (n = 24)	Log Brown Fat Volume		Log Brown Fat Volume normalized for LBM		Log SUV Mean	
	r	p	r	p	R	p
Age (years)	-0.04	0.86	-0.11	0.60	-0.01	0.95
BMI (kg/m^2^)	0.05	0.81	0.13	0.54	0.11	0.63
Duration of Amenorrhea (months)	-0.31	0.14*	-0.34	0.09	-0.46	0.02*
Activity (hours/week)	-0.37	0.10	-0.27	0.23	-0.18	0.44
Pulse (bpm)	0.42	**0.04**	0.39	0.07	0.14	0.50
Temperature (°F)	0.47	**0.02**	0.34	0.13	0.38	0.07
Percentage of Body Fat	0.41	**0.045**	0.38	0.06	0.31	0.14
Total Fat Mass (kg)	0.25	**0.24**	0.27	0.20	0.19	0.36
Total Lean Mass (kg)	-0.46	**0.02**	-0.10	0.60	-0.45	**0.03**
Resting Energy Expenditure (REE)	-0.19	0.43	0.01	0.90	-0.21	0.38
Irisin (mcg/ml)	0.42	**0.045**	0.39	0.07	0.30	0.17
TSH (microU/mL)	-0.15	0.50	0.02	0.90	-0.09	0.66
Total T3 (ng/ml)	0.62	**0.002**	0.58	0.003	0.65	**0.001**
Free T4 (ng/L)	0.62	**0.002**	0.43	0.04	0.51	**0.01**

*Spearman’s correlations was used for non parametric distributions

SUV mean: mean standardized uptake value.

### Relationship between Body Composition and Energy Expenditure with Brown Fat Measures

BAT volume correlated positively with percent body fat and inversely with lean mass (Table 2). Furthermore, mean SUV (BAT activity) correlated inversely with lean mass. No associations were observed between BAT volume or activity and resting energy expenditure. However, after adjusting for lean mass and heart rate (because lean mass and sympathetic drive (reflected in the heart rate) are known to influence REE as well as BAT), we found a positive association of BAT volume with REE (β estimate = 1.36, p = 0.047) supporting the fact that BAT is independently associated with REE.

### Relationships between Biochemical Parameters and Brown Fat Measures

**Fig 3:**
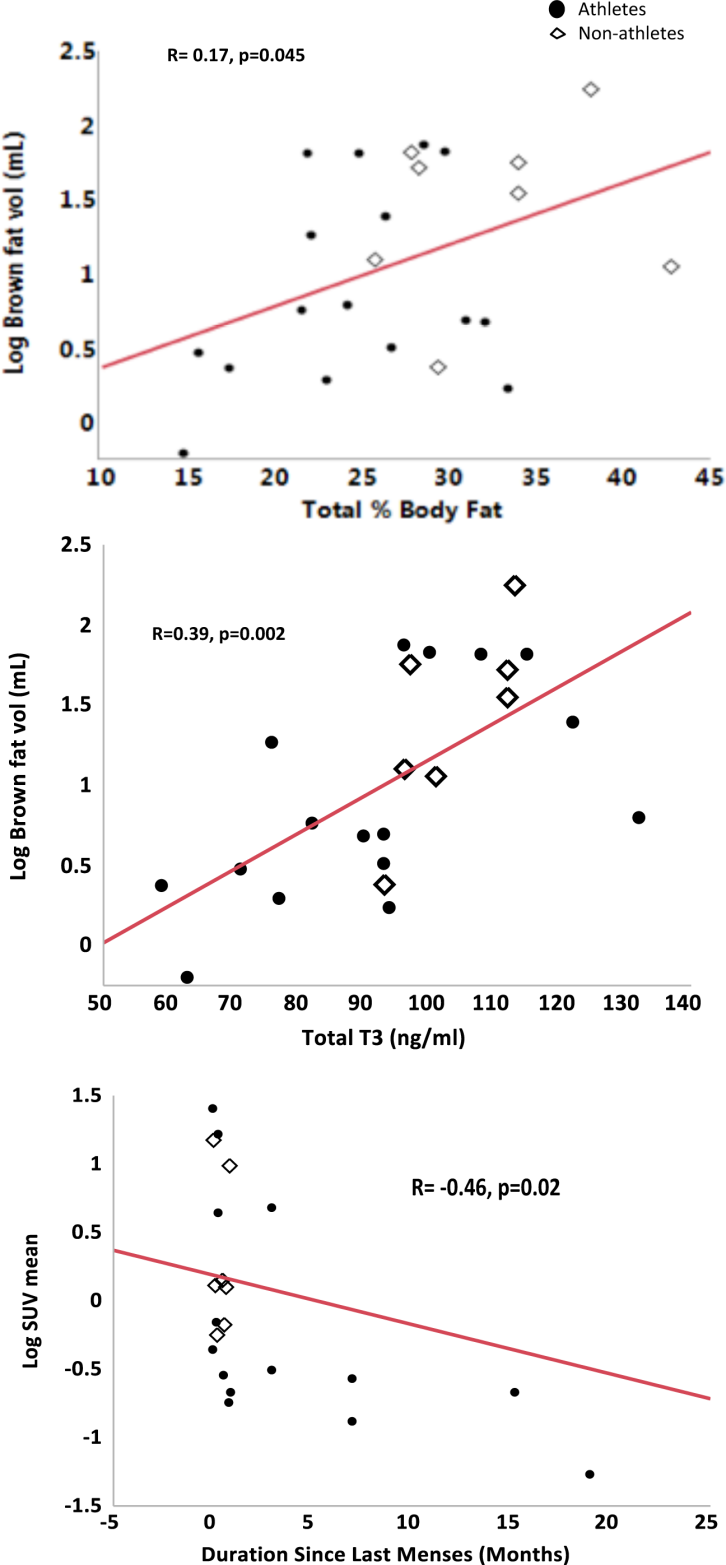
Linear associations of Brown Fat Volume with Clinical and Biochemical Characteristics. Positive associations were noted between brown fat volume and percent body fat in all subjects taken together, and with total T3 in all subjects and within athletes. There was a trend towards an inverse association between brown fat volume and lean body mass.

BAT volume was positively associated with irisin (Table 2). In addition, BAT volume and activity were positively associated with total T3 and free T4. After controlling for percent body fat (as a surrogate for leptin levels, as leptin increases T3 production as well as stimulates glucose uptake in BAT), BAT volume and activity continued to show a positive association with total T3 (β = 0.02, p = 0.01, and β = 0.03, p = 0.03 respectively) suggesting a positive association of thyroid hormones with BAT independent of fat mass (Fig 3).

## Discussion

There is increasing evidence that the impact of brown fat on energy homeostasis and non-shivering thermogenesis persists beyond the neonatal period in humans. Although the amount of brown fat in adult humans is small, its potential contribution to cold-induced thermogenesis is significant, with the capacity to contribute more to energy expenditure than even skeletal muscle, as shown in a rodent model[19]. Brown fat is increasingly being evaluated as a potential modifier of energy expenditure, and is reported to be present in lower quantities in obese compared with normal weight individuals [2]. Cao et al showed a possible mechanism of increased browning of white fat in mouse model by increased expression of hypothalamic brain-derived neurotrophic factor (BDNF), thereby leading to increased energy expenditure[20]. It is also known that endurance training in rats modestly increases browning of visceral fat [21]. On the other hand, studies have reported lower BAT activity in adult women with anorexia nervosa (a state of severe energy deficit and hypogonadism associated with physiological changes in many other endocrine axes including the growth hormone-IGF-1 axis and hypothalamic-pituitary-adrenal axis), suggesting an adaptive response to conserve energy [8].

The effect of chronic exercise on BAT is less clear with reports of increased BAT after habitual physical activity in cancer patients[6], but decreased BAT in male endurance athletes [7]. In our study of normal-weight athletes and non-athletes, we demonstrate that BAT measures trend lower in athletes compared with non-athletes, consistent with studies in anorexia nervosa and athletic men [7, 11], but in contrast to studies in cancer patients engaging in habitual physical activity[6]. The conflicting reports of the effect of exercise on BAT may stem from the degree and duration of exercise in participants of these studies, with the male endurance athletes being more similar to our study population than cancer patients undergoing routine exercise activity (but not endurance training).

Our findings are partially supported by a recent study in a rodent model that showed that exercise suppresses the thermogenic capacity of interscapular and aortic BAT but increases the ‘beiging’ of subcutaneous fat [22]. In our study we did not examine peripheral subcutaneous adipose tissue for browning or ‘beiging’ given that this requires biopsies for accurate quantification and is difficult to justify in young, healthy women in a research setting. However, based on previous manuscripts, ‘brown’ adipocytes are mostly found in the neck [23] and ‘beige’ adipocytes in the supraclavicular region [24]. Some functional thermogenic beige adipogenesis is also found in human neck fat [25]. In our subjects, both regions showed uptake of FDG.

Although, we did not find significant differences in BAT measures in oligo-amenorrheic compared with eumenorrheic athletes, we did find a negative association of BAT volume with duration of amenorrhea, an indicator of chronic estrogen deficiency and a state of energy deficit in athletes[9]. This suggests a likely effect of hypoestrogenism on BAT status, and is supported by up-regulation of BAT inducer, BMP8 by estrogen in rodents [5].

We did not find the expected associations of BAT measures with age and BMI, likely because the range of distribution of age and BMI in our study was too narrow to show significant correlations. However, BAT measures did correlate positively with percent body fat, a surrogate measure of energy stores and leptin [17, 26].This suggests that brown fat activation decreases when percent fat mass decreases, likely in an adaptive effort to conserve energy. This adaptation may also explain the unexpected inverse association we observed of lean mass with BAT volume and activity as in our cohort lean mass was negatively associated with fat mass. Also, leptin (secreted by adipose tissue) is known to increase T3 production and stimulate glucose uptake in BAT [27]. Thus, in conditions of energy deficit, lower leptin levels should correlate with lower total T3 levels and lower BAT activity. In our study, we found positive associations of thyroid hormone levels with BAT volume and activity. Interestingly, these associations persisted even after controlling for percent body fat, suggesting additional independent effects of thyroid hormones on BAT volume and activity, consistent with other studies [12]. We did not assess IL-6 levels, another BAT inducer in our study participants. Of note, however, Vosselman et al. [7] found no difference in IL-6 levels in endurance-trained compared with lean sedentary men. We did assess irisin levels in our participants, and similar to Bostrom et al.[28], we found the expected positive association of BAT measures with irisin levels, further validating our brown fat evaluations.

Among other factors, resting energy expenditure (REE) is affected by both lean mass and BAT volume. Athletes have a higher proportion of lean mass, which should increase their REE. Indeed, in our study, percent lean mass and REE trended higher in athletes than in non-athletes. Yet, body temperature was lower in athletes, likely attributable to lower BAT resulting in lower rates of non-shivering thermogenesis and hence lower body temperatures (despite higher REE).

A limitation of the study was the relatively small number of participants; however, our estimated sample size to detect a significant difference between athlete and non-athlete groups was based on robust power calculations. Of note, based on the observed effect size and post-hoc power calculations, a total of 450 subjects are necessary to detect a significant difference between the oligo-amenorrheic and eumenorrheic athlete groups, not feasible in a preliminary study. Our sample size did limit our ability to control for multiple comparisons in the correlational analyses. Another limitation is that participants were enrolled throughout the year, and there could be variability in brown fat activation depending on the season. However since all participants were exposed to cooling, the acute effects of ambient temperature were mitigated. Additionally, the proportion of subjects evaluated during specific seasons did not differ across groups. Another issue is the difficulty in characterizing chronic estrogen deficiency in humans. We used duration of amenorrhea (time since the first day of the last menstrual cycle) as an indication of chronic estrogen deficiency. However, this underestimates the chronicity of estrogen deficiency in some women as it does not capture periods of amenorrhea between occasional menses in women with irregular menstrual cycles. It is still a better measure of chronic estrogen deficiency than single estradiol level, which varies across the menstrual cycle in normally cycling women. Finally, the threshold used in the study has the possibility of overestimating brown fat volume by including white fat, as well as potentially causing balanced informational error, which if anything should dilute the difference between the groups.

Our study demonstrates a trend towards decreased brown fat in athletes compared to non-athletes and positive associations of brown fat with body fat and triidothyronine levels, and inverse associations within athletes with duration of amenorrhea, suggesting that activated brown fat can be modified in conditions of increased physical activity and hypoestrogenism, possibly to conserve energy. Larger prospective studies are necessary to further define the role of brown fat in modifying energy status in normal-weight athletes.
