# Correction: Effect of Chronic Athletic Activity on Brown Fat in Young Women

**DOI:** 10.1371/journal.pone.0160129

**Published:** 2016-08-10

**Authors:** Vibha Singhal, Giovana D. Maffazioli, Kathryn E. Ackerman, Hang Lee, Elisa F. Elia, Ryan Woolley, Gerald Kolodny, Aaron M. Cypess, Madhusmita Misra

There is an error in the byline. The third author’s name is spelled incorrectly. The correct name should be: Kathryn E Ackerman.
